# Kalman Filtering for Genetic Regulatory Networks with Missing Values

**DOI:** 10.1155/2017/7837109

**Published:** 2017-07-26

**Authors:** Qiongbin Lin, Qiuhua Liu, Tianyue Lai, Wu Wang

**Affiliations:** ^1^College of Electrical Engineering and Automation, Fuzhou University, Fuzhou, Fujian 350116, China; ^2^Fujian Key Lab of Medical Instrument and Pharmaceutical Technology, Fuzhou, Fujian 350116, China

## Abstract

The filter problem with missing value for genetic regulation networks (GRNs) is addressed, in which the noises exist in both the state dynamics and measurement equations; furthermore, the correlation between process noise and measurement noise is also taken into consideration. In order to deal with the filter problem, a class of discrete-time GRNs with missing value, noise correlation, and time delays is established. Then a new observation model is proposed to decrease the adverse effect caused by the missing value and to decouple the correlation between process noise and measurement noise in theory. Finally, a Kalman filtering is used to estimate the states of GRNs. Meanwhile, a typical example is provided to verify the effectiveness of the proposed method, and it turns out to be the case that the concentrations of mRNA and protein could be estimated accurately.

## 1. Introduction

According to the genetic central dogma, a specific protein can be generated by a complex gene expression process (including transcription process, translation process, and other interaction process) among DNAs, RNAs, and gene products [1, 2]. To guide the gene expression correctly, each stage of the gene expression should be regulated. The regulation functions for each stage form genetic regulatory networks (GRNs). Cleary, gene expression levels can be determined by GRNs. For this, a lot of GRNs models have been built to track the concentration of mRNA and protein, like Boolean model [3, 4], Bayesian model [5–7], differential equation model [8–11], and state-space model [12, 13]. However, due to the uncertainties of the system, time-varying delays [14–16] and data missing [17, 18] in real gene expression process, the measurements obtained from the sensor are usually contaminated by noise and cannot represent the true values well. Thus, a lot of filtering methods are proposed to reveal the true values.

In studying the stability of genetic regulatory networks, noise disturbances are one of the main factors that cannot be ignored, and it is mainly composed of process noise and measurement noise. In order to restrain these noise disturbances, many filtering methods like *H*_*∞*_ filter [19] and Kalman filter [20] are proposed to obtain stable GRNs. Although process noise and measurement noise were usually taken into consideration, the correlation between process noise and measurement noise always is ignored in these methods, so it does not have the generality from this point of view. In this paper, in order to make the filtering method more representative, the correlation between process noise and measurement noise would be taken into consideration; meanwhile, the correlation will also be decoupled in theory.

Generally, gene expression levels (the concentration of mRNA and protein) can be measured by the DNA microarray technology, but there are many reasons which can cause value miss like dust or scratch on the slide, inappropriate thresholds in preprocessing, insufficient resolution of the microarray, experimental errors during the laboratory processes, or image corruption [18]. So, the measured value for gene expression levels would contain a certain degree of distortion that would cause concentration value deviating from real concentration. To overcome this drawbacks, the set-values filtering for GRNs with missing value was proposed in [17, 21]; although this method has dealt with the specific well, it did not give a detailed explanation about missing value in a detailed mathematical formula, so, in this paper, the observation model with missing value will be given; meanwhile, a Kalman filtering will also be designed to obtain stable GRNs with missing value.

In this paper, an estimation problem for a class of discrete-time GRNs model with time-varying delays, missing values, and correlation of noise is considered. The rest of the paper is organized as follows. In Section 2, a discrete model of genetic regulation networks is introduced; we also built observation model with missing value to give a detailed explanation about it in mathematical formula; meanwhile, the correlation between process noise and measurement noise is decoupled in theory. In Section 3, a Kalman filtering is designed to estimate the real concentrations of GRNs; meanwhile, the stability of Kalman filtering is analyzed. In the Section 4, a typical example is provided to illustrate the effectiveness of the proposed method.

## 2. Problem Formulation

Clearly, a discrete-time model of genetic regulatory networks (GRNs) can be described as follows [21–23]:

(1) Mk+1=AkMk+BkfNk−σ+Z,Nk+1=CkNk+DkMk−σ,

where the descriptions of system's parameters are shown in Table 1.

In addition, *f*(·) ∈ *ℝ* is a monotonic function in Hill form, which represents the feedback regulation of the protein. Here, *f*_*i*_(*x*) = (*x*/*β*_*i*_)^*H*_*i*_^/(1 + (*x*/*β*_*i*_)^*H*_*i*_^), where *H*_*i*_ is the Hill coefficient and *β*_*i*_ is positive constant.

Let *M* ^*∗*^ and *N* ^*∗*^ denote the equilibrium points of system (1); define

(2) M¯k≜Mk−M∗,N¯k≜Nk−N∗.

Thus, system (1) can be rewritten as

(3) M¯k+1=AkM¯k+BkfNk−σ−fN∗,N¯k+1=CkN¯k+DkM¯k−σ.

Based on the first-order Taylor expansion, $f(N(k-\sigma ))-f(N^{*})={\left.\left(\partial f/\partial N\right)\right|}_{N=N^{*}}\bar{N}(k-\sigma )$, system (3) can be expressed as

(4) M¯k+1=AkM¯k+Bk∂f∂NN=N∗N¯k−σ,N¯k+1=CkN¯k+DkM¯k−σ.

In practice, the actual GRNs might be influenced by the dynamic reaction of the networks, time delays, and molecular noise. Based on system (4), discrete-time GRNs with observation equation and noises are considered:

(5) mk+1=A~kmk+B~kmk−σ+Fkwk,hk=Ekmk+vk,

where $m(k)≜{\left[\begin{array}{cc} \bar{M}(k{)}^{T} & \bar{N}(k{)}^{T} \end{array}\right]}^{T}$, *h*(*k*) ∈ *ℝ* ^*n*^ is the sampled output, *v*(*k*) is the external noise, *w*(*k*) is the process noise, *F*(*k*) is the noise driven matrix, and *E*(*k*) is the observation matrix. In addition,

(6) A~k≜Ak00Ck,B~k≜0Bk∂f∂NN=N∗Dk0.

Then, in order to solve the time-delay of the system (5), a new state vector is defined as follows:

(7) $$\begin{array}{c} x\left(\;k\;\right)≜{\left[\;\begin{array}{cccc} m^{T}\left(k\right) & m^{T}\left(k-1\right) & \cdots & m^{T}\left(k-\sigma \right) \end{array}\;\right]}^{T}. \end{array}$$

Using the new state variable (7) gives

(8) xk+1=Φkxk+Γkw¯k,zk=Hkxk+v¯k,

where $\bar{w}(k)$ and $\bar{v}(k)$ are white, zero-mean, correlated noises; furthermore,

(9) Φk=A~k0⋯0B~kIn0⋯000In⋯00⋮⋮⋱⋮⋮00⋯In0n×σ×n×σ,w¯k=wTk0⋯0n×σ×1T,v¯k=vk,Γk=diag⁡Fk0⋯0n×σ×n×σ.

As for the measurements model with missing value, it can be expressed as that measurement values lost at a certain probability, so, the measurements model with missing value can be described as follows [24]:

(10) $$\begin{array}{c} y\left(\;k\;\right)=\xi \left(\;k\;\right)z\left(\;k\;\right)+\left(\;1-\xi \left(\;k\;\right)\;\right)y\left(\;k-1\;\right), \end{array}$$

where *y*(*k*) is received by the estimator, the initial state *x*(0) is independent of *ξ*(*k*), $\bar{w}(k)$, and $\bar{v}(k)$ and satisfies the fact that *𝔼*[*x*(0)] = *μ*_0_, *𝔼*[(*x*(0) − *μ*)(*x*(0) − *μ*)^*T*^] = *P*_0_, and *ξ*(*k*) ∈ *ℝ* obey the Bernoulli distribution, and it is uncorrelated with other random variables. There are two basic properties about *ξ*(*k*):

(11) Probξk=1=Eξk≔αk,Probξk=0=1−Eξk≔1−αk,

where 0 ≤ *α*(*k*) ≤ 1. If *α*(*k*) = 0, it means the measurements value is lost at *k*, and there is no missing value with *α*(*k*) = 1. More properties about the distribution of *ξ*(*k*) are showed in [24].

Then, substituting the observation equation of system (8) into (10), thus, a discrete-time model of GRNs with the observation equation with missing value is established as follows:

(12) Xk+1=Φ~kXt+Γ~kWk,yk=H~kXk+ξkv¯k,

where

(13) Xk=xkyk−1,Wk=w¯tv¯t,Φ~k=Φk0ξkHk1−ξkIm,Γ~k=Γk00ξkIm,H~k=ξkHk1−ξkIm.

Let *Q*_*w*_(*k*) denote the autocovariance matrix of $\bar{w}(k)$, *Q*_*v*_(*k*) denote the autocovariance matrix of $\bar{v}(k)$, and *S*(*k*) denote the cross-covariance matrix of $\bar{w}(k)$ and $\bar{v}(k)$.

For (12), there is some statistical information:

(14) Wk~N0,QWk,v¯k~N0,Qvk,

where

(15) QWkEw¯kw¯Tkw¯kv¯Tkv¯kw¯Tkv¯kw¯Tk=QwkS1kS1TkQvk,SkEw¯kv¯kv¯Tk=Ew¯kv¯Tkv¯kv¯Tk=S1TkQvk

and where $S_{1}=\bar{w}(k){\bar{v}}^{T}(k)$.

To simplify the calculation, $\tilde{\Phi }(k)$, $\tilde{\Gamma }(k)$, and $\tilde{H}(k)$ can be broken down into some simple separations as follows:

(16) Φ~kΦk0ξkHk1−ξkIm=Φk0αkHk1−αkIm+ξk−αk00Ht−Im≜Φ0k+ξk−αkΦ1k,Γ~kΓk00ξkIm=Γk00αkIm+ξk−αk000Im≜Γ0k+ξk−αkΓ1k,H~kξkHk1−ξkIm=αkHk1−αkIm+ξk−αkHt−Im≜H0k+ξk−αkH1k.

Since the process noises of this system are correlated with the observation noises, to decouple the relevance about $\bar{w}(k)$ and $\bar{v}(k)$, according to system (12), $y(t)-\tilde{H}(k)X(k)-\xi (k)v(k)=0$; obviously,

(17) $$\begin{array}{c} J\left(\;k\;\right)\left(\;y\left(\;t\;\right)-\tilde{H}\left(\;k\;\right)X\left(\;k\;\right)-\xi \left(\;k\;\right)v\left(\;k\;\right)\;\right)=0 \end{array}$$

and then adding (17) to the state equation of (12), we have

(18) Xk+1Φ~kXk+Γ~kWk+Jkyt−H~kXk−ξkvk=Φ~k−JkH~kXk+Jkyk+Γ~kWk−Jkξkvk,

where *J*(*k*) ∈ *R* ^*n*×*m*^. Clearly, the last two terms in (18) are the process noises

(19) Φ∗k=Φ~k−JkH~k,W∗k=Γ~kWk−Jkξkvk.

Since Kalman filtering requires that the process noise and the measurement noise must be white uncorrelated Gaussian noise, then consider the correlation between process noise and measurement noise firstly:

(20) $$\begin{array}{c} E\left[\;W^{*}\left(\;k\;\right)v^{T}\left(\;k\;\right)\;\right]=\Gamma_{0}\left(\;k\;\right)S_{1}\left(\;k\;\right)-\alpha \left(\;k\;\right)J\left(\;k\;\right)Q_{v}\left(\;k\;\right). \end{array}$$

Let *𝔼*[*W* ^*∗*^(*k*)*v* ^*T*^(*k*)] = 0, and then *J*(*k*) is

(21) $$\begin{array}{c} J\left(\;k\;\right)=\alpha^{-1}\left(\;k\;\right)\Gamma_{0}\left(\;k\;\right)S_{1}\left(\;k\;\right)Q_{v}^{-1}. \end{array}$$

Clearly, if *J*(*k*) is chosen as (21), *W* ^*∗*^(*k*) and $\bar{v}(k)$ are uncorrelated.

Secondly, we discuss *W* ^*∗*^(*k*),

(22) EW∗k=Γ~kEWk−αkJkEvk=0,EW∗kW∗Tt=covW∗k,W∗Tt=varW∗kδkt,var⁡W∗k=Q∗=Γ0QwΓ0T−αkΓ0SkJTk+αk1−αkΓ1kQwΓ1Tk−αk1−αkΓ1kSkJTk−αkJkSTkΓ0Tk−αk1−αkJTkSTkΓ1Tk+αkJkQvJTk

so if *J*(*k*) = *α* ^−1^(*k*)Γ_0_(*k*)*S*_1_(*k*)*Q*_*v*_^−1^, *W* ^*∗*^(*k*) is a white, zero-mean noise.

## 3. Main Results

In this section, the Kalman filtering is designed for obtaining the minimum variance estimation. Firstly, the expression of the filtering error *P* is calculated, and then the Kalman gain *K* can be obtained by minimizing the covariance matrix of the filtering error *P*; at last, the recursion of the filtering error *P* is calculated; thus, the design of Kalman filtering is completed.

According to system (12) and (18), the state prediction equation can be calculated as

(23) X^k+1 ∣ k=Φ~kX^k ∣ k+Jkyk−H~kX^k ∣ k

and the measurement update equation is

(24) $$\begin{array}{c} \hat{y}\left(\;k+1\;\right)=\tilde{H}\left(\;k+1\;\right)\hat{X}\left(\;k+1\;|\;k\;\right). \end{array}$$

So, the optimal state estimation is

(25) X^k+1 ∣ k+1=X^k+1 ∣ k+Kk+1yk+1−y^k+1,

where *K*(*k* + 1) denotes the Kalman gain.

Then, the posterior estimation error can be computed as follows:

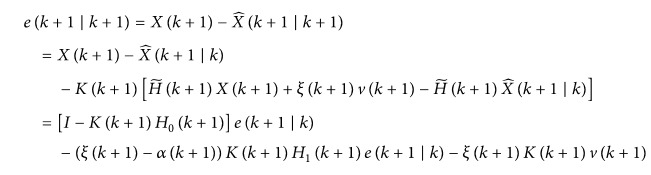
(26)and the covariance matrix of estimation error can be described as

(27) Pk+1 ∣ k+1=Eek+1 ∣ k+1eTk+1 ∣ k+1.

Substituting (26) into (27) gives

(28) Pk+1 ∣ k+1=Eek+1 ∣ k+1eTk+1 ∣ k+1=I−Kk+1H0k+1Pk+1 ∣ k·I−Kk+1H0k+1T+αk+1·1−αk+1Kk+1H1k+1Pk+1 ∣ k·H1Tk+1+αk+1Kk+1QvKTk+1=Pk+1 ∣ k−Kk+1H0k+1Pk+1 ∣ k−Pk+1 ∣ kKk+1H0k+1T+Kk+1·H0k+1Pk+1 ∣ kH0Tk+1+αk·1−αkH1k+1qk+1H1Tk+1+αk+1Qvk+1KTk+1.

Then, *L* is designed to minimize *P*(*k* + 1/*k* + 1), and

(29) L=Pk+1 ∣ kH0Tk+1·H0k+1Pk+1 ∣ kH0Tk+1+αk1−αkH1k+1qk+1H1Tk+1+αk+1Qvk+1−1H0k+1Pk+1 ∣ k;

thus, *P*(*k* + 1/*k* + 1) can be rewritten as
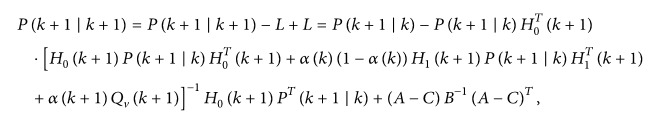
(30)where 
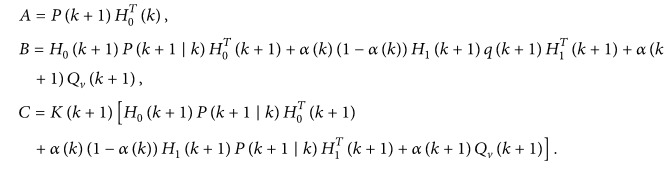
(31)Let *A* = *C*; the covariance matrix of estimation error is minimized. Thus 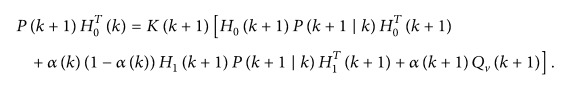
(32)Furthermore,
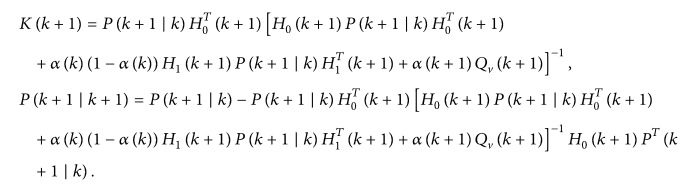
(33)


According to (18) and (25), the estimation error $\tilde{X}(k+1/k)$ can be obtained

(34) X~k+1 ∣ k=Xk+1−X^k+1 ∣ k=Φ~kXk−JkH~kXk+Jkyk+W∗−Φ~kX^k ∣ k=Φ0k−JkH0kX~k+ξk−αkΦ1k−JkH1kX~k+W∗;

thus

(35) Pk+1 ∣ k=EX~k+1 ∣ kX~Tk+1 ∣ k=Φ0k−JkH0kPk ∣ k·Φ0k−JkH0kT+ξk−αk·Φ1k−JkH1kPk ∣ k·Φ1k−JkH1kT+Q∗.

The linear optimal filtering, (23), (25), (33), and (35), is uniformly asymptotically stable when the linear discrete-time-varying stochastic system (12) is uniformly controllable and observable [24].

## 4. Numerical Example

In this section, an example will be provided to show the effectiveness of the proposed method. In* Escherichia coli* [25], the dynamics of the networks have been experimentally studied, and the model of 3-gene repressilator is given as follows:

(36) M˙i=−Mi+αi1+NjH,N˙i=−βiNi+γiMi,

where *M*_*i*_ denotes the concentrations of three mRNA and *N*_*i*_ denotes the concentrations of three repressor-proteins, *α*_*i*_ is the feedback regulation coefficient, *β*_*i*_ denotes the ratio of the protein decay rate to the mRNA, and *H* is the Hill coefficient, *i* = lacl, tetR, cl; *j* = cl, lacl, tetR.

The discrete-time GRNs model based on the method in [26] can be obtained as

(37) Mik+1=e−hMik+1−e−hαi1+NjHk−σ,Nik+1=e−βihNik+1−e−γihMik−σ.

Let *h* = 1, the Hill coefficient *H* = 2, the time-delay *σ* = 1, *f*(*x*) = *x* ^2^/(1 + *x* ^2^), and the other parameters are taken as follows:

(38) α1=1.2656,α2=0.6328,α3=1.4238,β1=β2=β3=0.6703,γ1=0.6,γ2=0.4,γ3=0.5,F=diag⁡0.2,0.3,0.2,0.3,0.2,0.4.

So, the parameters of system (4) can be obtained:

(39) A=0.36790000.36790000.3679,B=1−e−h00−α1−α2000−α30,C=β1000β2000β3,D=γ1000γ2000γ3.

**Figure 1:**
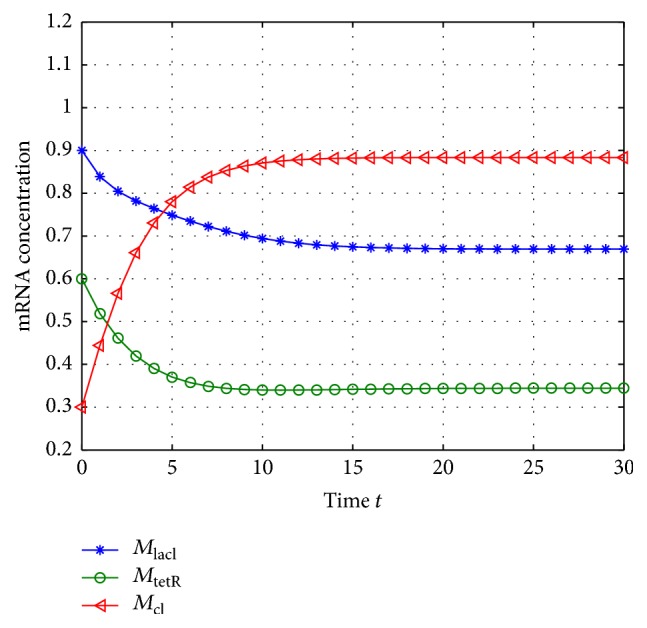
The concentration of mRNA with *w*(*t*) = 0.

**Figure 2:**
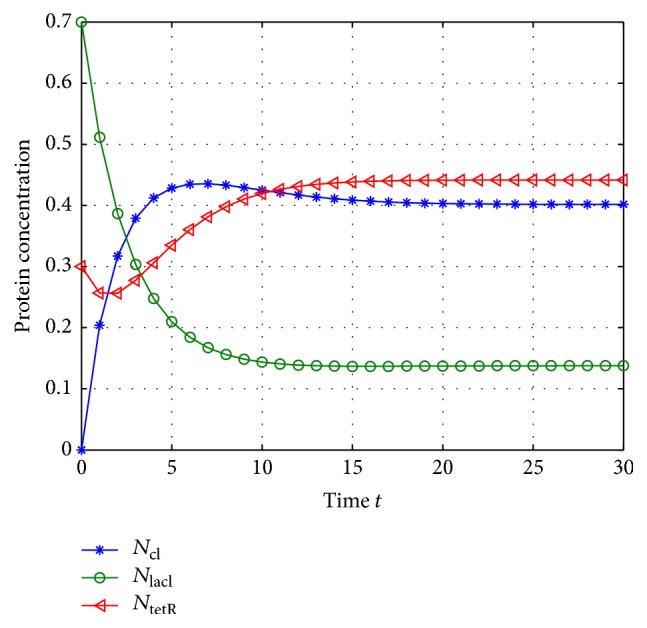
The concentration of protein with *w*(*t*) = 0.

According to system (3), we can get that the mRNA and proteins will adjust each other; they will also degrade along with the time, so the GRNs would tend to be equilibrium if there are no noise disturbances, and the unique equilibrium can be checked easily when *w*(*t*) = 0; thus, the system's states *M*(*k*) and *N*(*k*) with *w*(*t*) = 0 are shown in Figures 1 and 2.

From Figures 1 and 2, we can get that the states of the GRNs stay at a point stably, so the equilibrium can be calculated; that is,

(40) M∗=0.6695,0.3444,0.8833,N∗=0.4016,0.1378,0.4416.

Now, check the states of system (37) under the excitation of external disturbances; let the initial states be

**Figure 3:**
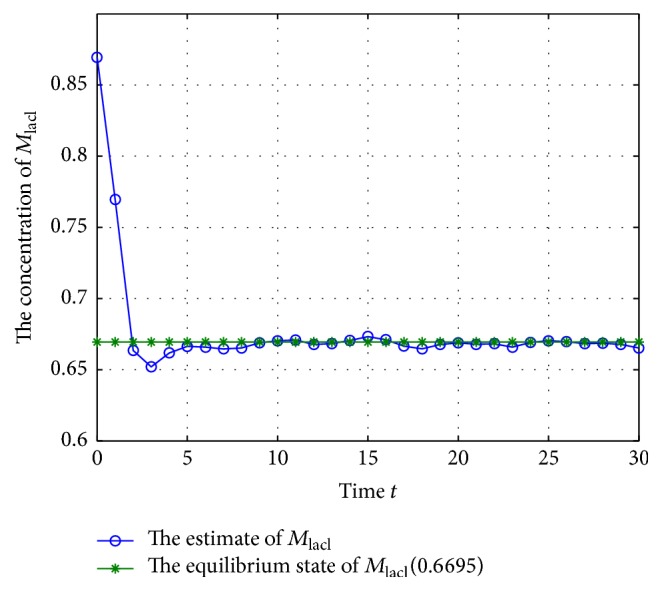
The trajectory of concentration of *M*_lacl_ (missing rate 10%).

**Figure 4 fig4:**
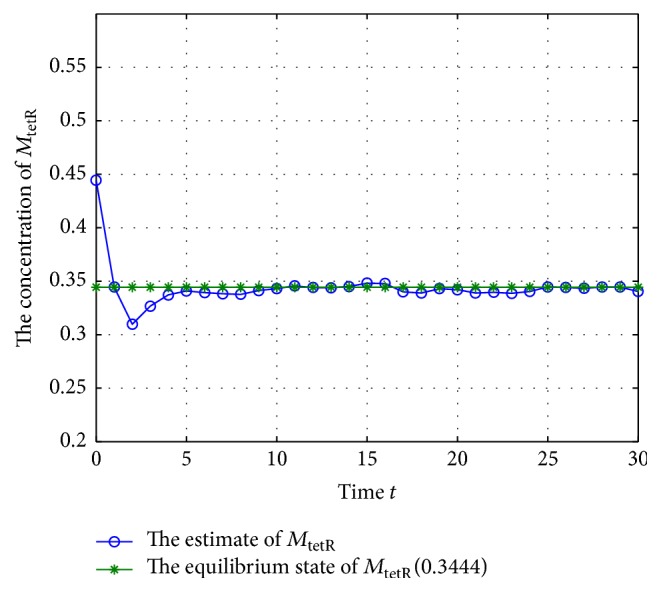
The trajectory of concentration of *M*_tetR_ (missing rate 10%).

**Figure 5 fig5:**
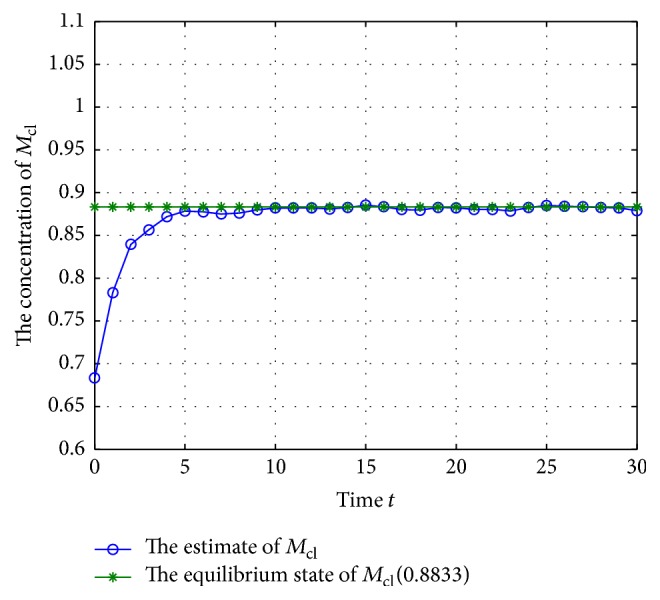
The trajectory of concentration of *M*_cl_ (missing rate 10%).

**Figure 6 fig6:**
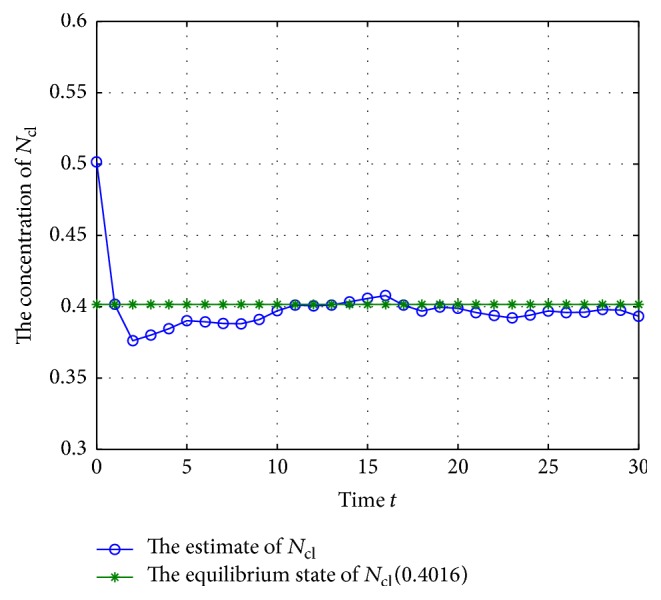
The trajectory of concentration of *N*_cl_ (missing rate 10%).

**Figure 7 fig7:**
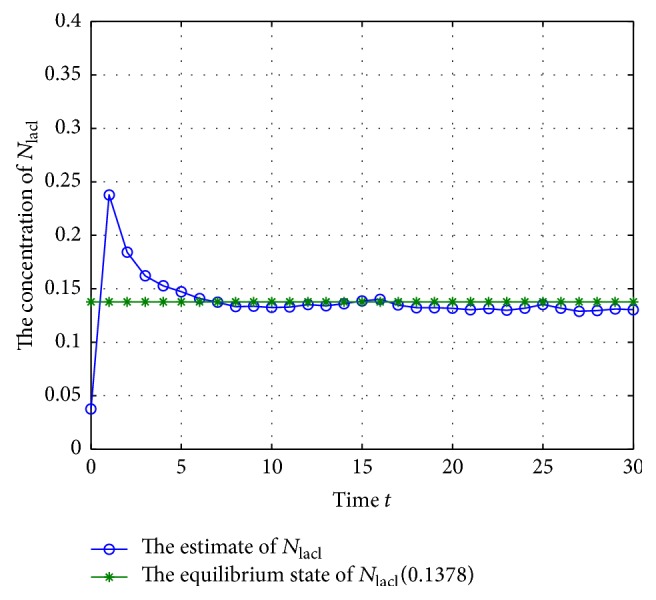
The trajectory of concentration of *N*_lacl_ (missing rate 10%).

**Figure 8:**
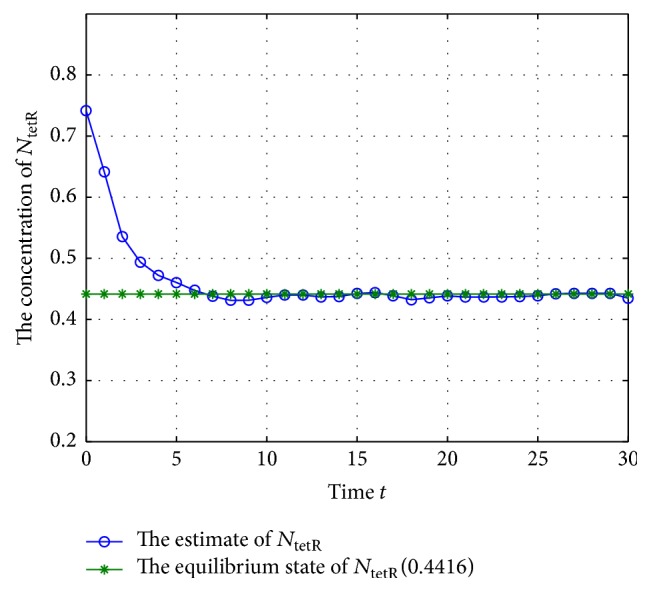
The trajectory of concentration of *N*_tetR_ (missing rate 10%).

(41) M−1=0.8695,0.4444,0.6833T,M0=0.7695,0.3444,0.7833T,N−1=0.5016,0.0378,0.7416T,N0=0.4016,0.2378,0.6416T,

and *Q*_*w*_ = 12, *Q*_*v*_ = 36, and *𝔼*[*w*_*i*_ *v*_*j*_^*T*^] = 1.2 (where *i* = 1,2,…, *n*, *j* = 1,2,…, *n*); the estimate values of the concentration of mRNA and proteins are shown in Figures 3 [Fig fig4] [Fig fig5] [Fig fig6] [Fig fig7]–8.

According to Figures 3 [Fig fig4] [Fig fig5] [Fig fig6] [Fig fig7]–8, the blue lines show the estimate values of mRNAs and protein, and the green lines illustrate the equilibrium of GRNs; we can get that the concentration of mRNAs and protein tends to the equilibrium well under the excitation of external disturbances, so, the Kalman filtering designed in this paper is effective for the GRNs with missing value and noise correlation.

In order to test out the influence of the missing rate, the experiments with four missing rates of 10%, 20%, 30%, and 50% are carried out. In addition, the normalized root mean squared error (NRMSE) [27] is used to indicate the influence level of the missing rate, and the NRMSE is defined as

(42) $$\begin{array}{c} NRMSE=\sqrt{\frac{mean{\left({\hat{x}}_{k}-x_{k}\right)}^{2}}{mean\;x_{k}^{2}}}. \end{array}$$

So, the NRMSE are shown in Table 2.

Compared with the NRMSE obtained by set-membership filtering given in [17], in spite of the missing rate increases from 10% to 30%, the NRMSE listed in Table 2 increases slightly; however, the NRMSE increases greatly with the increasing of the missing rate in [17]. Moreover, at the low level of missing rate, the set-membership filtering has a better performance, but at the high level of missing rate, the method proposed in this paper is more appropriate than the set-membership filtering, and the cut-off point roughly equals 14.66%. Thus, it shows that the proposed method is more effective for the filtering problem for GRNs.

## 5. Conclusion

In this paper, a discrete model of genetic regulation networks is introduced; we also built an observation model with missing value to give a detailed explanation about it in mathematical formula; meanwhile, the correlation between process noise and measurement noise is decoupled in theory. Finally, a Kalman filtering is designed to obtain stable GRNs; meanwhile, the simulation result shows that the method proposed in this paper is effective for the GRNs with missing value, and compared with the set-membership filtering, the Kalman filtering has a better performance when the missing rate stays at a high level.

## Figures and Tables

**Table 1:** The parameter descriptions of system (1).

Parameter	Description
*M*(*k*)	The concentrations of mRNA
*N*(*k*)	The concentrations of protein
*A*(*k*)	The degradation rates of mRNA
*C*(*k*)	The degradation rates of protein
*B*(*k*)	The coupling coefficient of the genetic networks
*D*(*k*)	The translation rate
*Z*	The bounded constant which denotes the dimensionless transcriptional rate [21]

**Table 2:** The average values of NRMSE.

Method	Method	
	Kalman	Set-membership [17]
10%	0.5389	0.4125
20%	0.5785	0.7074
30%	0.5800	—
50%	0.6824	0.8792
